# A transcriptomics approach uncovers novel roles for poly(ADP-ribosyl)ation in the basal defense response in *Arabidopsis thaliana*

**DOI:** 10.1371/journal.pone.0190268

**Published:** 2017-12-28

**Authors:** Amy G. Briggs, Lori C. Adams-Phillips, Brian D. Keppler, Sophia G. Zebell, Kyle C. Arend, April A. Apfelbaum, Joshua A. Smith, Andrew F. Bent

**Affiliations:** 1 Department of Biology, Beloit College, Beloit, WI, United States of America; 2 Department of Plant Pathology, University of Wisconsin–Madison, Madison, WI, United States of America; Indiana University, UNITED STATES

## Abstract

Pharmacological inhibition of poly(ADP-ribose) polymerase (PARP) or loss of *Arabidopsis thaliana PARG1* (poly(ADP-ribose) glycohydrolase) disrupt a subset of plant defenses. In the present study we examined the impact of altered poly(ADP-ribosyl)ation on early gene expression induced by the microbe-associate molecular patterns (MAMPs) flagellin (flg22) and EF-Tu (elf18). Stringent statistical analyses and filtering identified 178 genes having MAMP-induced mRNA abundance patterns that were altered by either PARP inhibitor 3-aminobenzamide (3AB) or *PARG1* knockout. From the identified set of 178 genes, over fifty Arabidopsis T-DNA insertion lines were chosen and screened for altered basal defense responses. Subtle alterations in callose deposition and/or seedling growth in response to those MAMPs were observed in knockouts of At3g55630 (*FPGS3*, a cytosolic folylpolyglutamate synthetase), At5g15660 (containing an F-box domain), At1g47370 (a TIR-X (Toll-Interleukin Receptor domain)), and At5g64060 (a predicted pectin methylesterase inhibitor). Over-represented GO terms for the gene expression study included "innate immune response" for elf18/*parg1*, highlighting a subset of elf18-activated defense-associated genes whose expression is altered in *parg1* plants. The study also allowed a tightly controlled comparison of early mRNA abundance responses to flg22 and elf18 in wild-type Arabidopsis, which revealed many differences. The PARP inhibitor 3-methoxybenzamide (3MB) was also used in the gene expression profiling, but pleiotropic impacts of this inhibitor were observed. This transcriptomics study revealed targets for further dissection of MAMP-induced plant immune responses, impacts of PARP inhibitors, and the molecular mechanisms by which poly(ADP-ribosyl)ation regulates plant responses to MAMPs.

## Introduction

The plant immune system is composed of at least three basic components: pre-formed defenses, infection-induced basal defenses, and *R*-gene mediated defenses [1–3]. Once pre-formed structural barriers are breached, the basal immune response is activated by pattern recognition receptors (PRRs) such as Flagellin-Sensitive 2 (FLS2) and EF-TU Receptor (EFR). PRRs recognize specific ubiquitously-expressed, highly conserved microbe-associated molecular patterns (MAMPs) such as the bacterial flagellin or Elongation Factor-Tu (EF-Tu) proteins. The peptides flg22 and elf18, representing the core epitopes of flagellin and EF-Tu that are recognized by plant receptors, are widely used for research in place of the larger MAMPs. Activated basal defenses include cell wall reinforcement via callose deposition, production of ethylene and reactive oxygen species (ROS), activation of a mitogen-activated protein (MAP) kinase cascade, and elevated expression of defense-associated genes. Arabidopsis seedling growth inhibition in response to MAMPs is an additional phenotype used to quantitatively measure activation of basal defenses [4–6]. Previous studies have demonstrated that disrupting the post-translational modification poly(ADP-ribosyl)ation can disrupt many of these basal responses [7–12].

Poly(ADP-ribosyl)ation is a functional component of the plant response to biotic stress. The poly(ADP-ribose) polymerase (PARP) inhibitor 3-aminobenzamide (3AB) blocks MAMP-induced callose and lignin deposition, phenylalanine ammonia lyase (PAL) activation, and phenylpropanoid pigment accumulation. In addition, knocking out one of two expressed poly(ADP-ribose) glycohydrolase (PARG) genes in Arabidopsis (*PARG1*) exacerbates both MAMP-induced seedling growth inhibition and MAMP-induced pigment accumulation, and causes greater susceptibility to the necrotrophic pathogen *Botrytis cinerea* [7].

Poly(ADP-ribosyl)ation, carried out by poly(ADP-ribose) polymerases, is a common post-translational modification in multicellular eukaryotes [13,14]. PARPs use nicotinamide adenine dinucleotide (NAD^+^) as a substrate to catalyze the synthesis, attachment, and elongation of ADP-ribose polymers to target proteins. PARPs act as DNA damage sensors, since DNA nicks activate some of the most abundant PARP isoforms [15–17]. Activated PARP can also consume large amounts of NAD^+^ and significantly modulate overall cellular levels of NAD^+^ [15,18]. Activated PARP auto-modifies (poly(ADP-ribosyl)ates) itself as well as other nuclear proteins such as histones [19]. This modification can affect chromatin structure, transcription, replication, and DNA repair processes through PARP-mediated recruitment of other proteins [20–22]. PARP activity is also a marker of genotoxic stress responses and programmed cell death in animals. At the organismal level, poly(ADP-ribosyl)ation in animals contributes to the pathology of strokes, ischemia, heart attacks, and chemotherapy treatments [23–25]. Roles of poly(ADP-ribosyl)ation in plant responses to biotic and abiotic stress have also been established [7,10,12,26–29]. DNA damage is also active in infected plant tissues [27].

PARP inhibitors have long been used in animal systems to selectively inhibit PARPs, typically functioning as competitive inhibitors that mimic the nicotinamide moiety of NAD^+^ and disrupt the poly(ADP-ribosyl)ation of proteins. 3AB inhibits PARPs in animals [30–33] and in plants [10,34,35]. As is common in other multicellular eukaryotes, at least three putative PARPs are encoded by the Arabidopsis genome, PARP1 (At2g31320), PARP2 (At4g02390), and PARP3 (At5g22470) [36]. Pharmacological PARP inhibitors can therefore be used to overcome potential functional redundancies, and also allow conditional inactivation of PARP activity. In addition the impacts of 3AB on plant defense noted above, treatment of plants with 3AB or 3-methoxybenzamide (3MB) (another PARP inhibitor) can improve resistance to abiotic stresses such as high light and oxidative damage [10,37,38], inhibit differentiation of tracheary elements [39], protect plants from oxidative and heat shock induced programmed cell death [40,41], and inhibit oxidative stress-induced PAL activity [7,42].

Poly(ADP-ribosyl)ation is a reversible modification. PARG proteins cleaves the sugar backbones of ADP-ribose polymers, [43]. Although PARG activity can reverse the poly(ADP-ribosyl)ation of target proteins, it cannot restore the large amounts of NAD^+^ consumed by PARP, and it may also free PARP substrates for further poly(ADP-ribosyl)ation. PARG activity also can increase cellular pools of toxic, free ADP-ribose, a known cell death signal in mammalian cells [44,45]. Hence, PARG may either counteract or further contribute to the impacts of PARP activation, depending on cellular context [46].

PARG plays an important role in genotoxic stress responses in animals [47–49]. Known animal genomes, including rhesus monkey, cow, marmoset, mouse, human, chimpanzee, drosophila, and rat, encode only one *PARG* gene, and knocking out this single gene in *Drosophila* and mouse leads to accumulation of toxic ADP-ribose polymers and embryonic lethality [50,51]. Arabidopsis is thus a rare example of a eukaryote with two expressed *PARG* genes, which are present due to gene duplication (At2g31865 and At2g31870). Arabidopsis PARG1 has been implicated in circadian rhythm regulation [9], genotoxic stress responses, defense responses [7,52–54], cell division, and development [55].

*PARG1* (also known as *Tej)* was first identified in Arabidopsis in a mutant screen for alterations in circadian clock gene expression [9]. The *parg1* mutant showed accumulation of ADP-ribose polymers, a phenotype which could be abolished by treatment with PARP inhibitor, indicating that PARG1 does, in fact, act as a poly(ADP-ribose) glycohydrolase in Arabidopsis tissue. We have previously reported the biotic stress-related characterization of plants carrying a second or third mutant allele of *parg1*, both of which showed increased susceptibility to necrotroph infection and treatment with the DNA-crosslinking agent mitomycin-C treatment [7,56], and more recent studies have also demonstrated a protective role for both PARG1 and PARG2 in genotoxic stress [53]. *parg1* knockouts also exhibit over-activation of some basal defense responses in response to the MAMP elf18, including phenylpropanoid pigment accumulation and seedling growth inhibition [7,12].

The lack of known cellular pathways involving poly(ADP-ribosyl)ation in plants has meant that there are few implicated targets to examine after establishing that poly(ADP-ribosyl)ation impacts defense responses [54]. We therefore deployed a transcriptomics discovery approach to uncover specific cellular pathways (including but not limited to defense responses) that are impacted by PARP, PARG and poly(ADP-ribosyl)ation. We report here a genome-wide gene expression analysis of the effects of PARP inhibitor and, separately, *parg1* knockout, on early MAMP-induced gene expression in the plant basal defense response. We studied *Arabidopsis thaliana* wild-type (Col-0), 3AB-treated and *parg1-2* T-DNA knockout plants responding to the MAMP elicitors flg22 or elf18. We have uncovered previously unknown roles for folylpolyglutamate synthetase, a toll/interleukin-1 receptor (TIR)-domain containing protein, an F-box domain containing protein, and a pectin methylesterase inhibitor in plant basal defense responses. These results provide possible links between the activation of poly(ADP-ribosyl)ation and induced defense responses.

## Materials and methods

### Plant lines and growth conditions

*Arabidopsis thaliana* (accession Col-0) plants and homozygous *parg1* knockout (SALK_116088) seeds were surface sterilized, plated on solid 1/2X MS media + 1.5% (w/v) sucrose + 1X Gamborg’s vitamins (Sigma, St. Louis, MO, USA), stored at 4°C for 2 days to break dormancy, and then grown at 22°C under short-day conditions (9hr light/15hr dark; 100–150 μmol m^-2^ s^-1^). After 5 days, seedlings were then transferred to 24-well plates (4 seedlings per well) containing 400 μL 1/2X Murashige and Skoog salts (Sigma, St. Louis, MO, USA) + 1.5% (w/v) sucrose + 1X Gamborg’s vitamins media. One 24-well plate of seedlings corresponded to one treatment. Plates were assigned a number 1–12 and randomly placed in a 4 x 3 grid using random sequence generating software (Random.org).

### Treatment conditions

**Fig 1:**
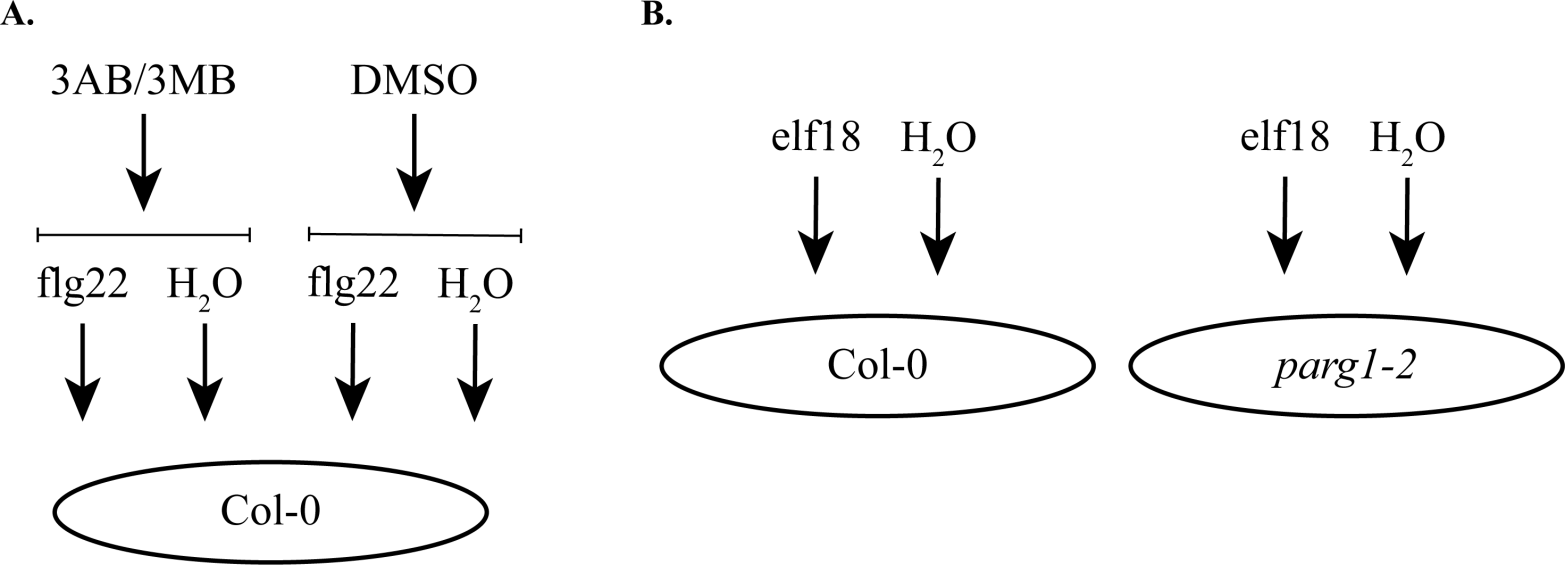
Experimental design. **A.** Three biological replicate pools of 48 ten day-old wild-type (Col-0) seedlings were pre-treated for two hours with either 3AB, 3-MB, or vehicle (DMSO), and then treated for one hour with either flg22 flagellin peptide or sterile H_2_O. **B.** Three biological replicate pools of 48 ten day-old wild-type (Col-0) or *parg1-2* knockout seedlings were treated for one hour with either elf18 EF-TU peptide or sterile H_2_O.

The experimental design included three replicates of nine treatments (Fig 1): 1. Col-0 (wild-type) untreated 1hr; 2. Col-0 + flg22 1hr; 3. Col-0+3AB 1hr; 4. Col-0+3MB 1hr; 5. Col-0 + flg22 3AB 1hr; 6. Col-0 + flg22 + 3MB 1hr; 7. Col-0 + elf18 1hr; 8. *parg1-2* untreated 1hr; and 9. *parg1-2* + elf18 1hr. Each biological replicate included 48 ten day-old Arabidopsis seedlings pooled for each treatment. Plants were pre-treated at two-minute intervals for one hour with either 4 μL 60% DMSO (untreated, no PARP inhibitor) or 4 μL 250mM 3AB (Sigma, St. Louis, MO, USA) (dissolved in 60% DMSO) or 250mM 3MB (Sigma, St. Louis, MO, USA) (dissolved in 60% DMSO). After pre-treatment, which was done to cause PARP inhibition prior to exposure to MAMPs, plates were gently agitated ten times while flat on a countertop to ensure equal mixing. One hour after pre-treatment [12], plants were then treated at two-minute intervals with either 4 μL ddH_2_O (untreated) or 4 μL 1mM flg22 (GenScript, Piscataway, NJ, USA) or elf18 (GenScript, Piscataway, NJ, USA) (final MAMP concentration = 1μM) with no further mixing. One hour after this treatment, seedlings were combined into two pools per treatment (48 seedlings per pool) and snap-frozen. An extra plate of Col-0 seedlings treated with flg22 and flg22+3AB was used to confirm blockage of callose by 3AB 24 hours after flg22 treatment. One additional plate each of Col-0 and *parg1-2* was used to confirm the presence of transgene and reduced *PARG1* mRNA in the *parg1-*2 plants compared to Col-0. The three biological replicates using this method were performed on separate dates.

### RNA isolation and cDNA synthesis

Total RNAs were isolated from one pool per treatment per biological replicate using QIAGEN RNeasy mini kit and treated with DNAse using the Qiagen RNase-Free DNAse set (Qiagen, Valencia, CA, USA). RNA quality and concentration was preliminarily verified by spectrophotometry and gel electrophoresis, and then sent to the Gene Expression Center (University of Wisconsin-Madison Biotechnology Center, Madison, WI, USA) for RNA integrity checks, double-stranded cDNA synthesis (NimbleGen Roche, Madison, WI, USA) and cDNA purification. cDNA was then submitted to NimbleGen Roche.

### Array hybridization and initial data analysis

cDNA was labeled with Cy3 and hybridized to a NimbleGen *Arabidopsis thaliana* 60-mer 4-plex 4x72k expression array (30,361 genes, 2 probes per target). Two technical replicates (two locations on one array) were performed. To generate expression data, quantile normalization [57] and robust multichip average (RMA) analysis [58] were performed (NimbleGen Roche, Madison, WI, USA).

**Fig 2:**
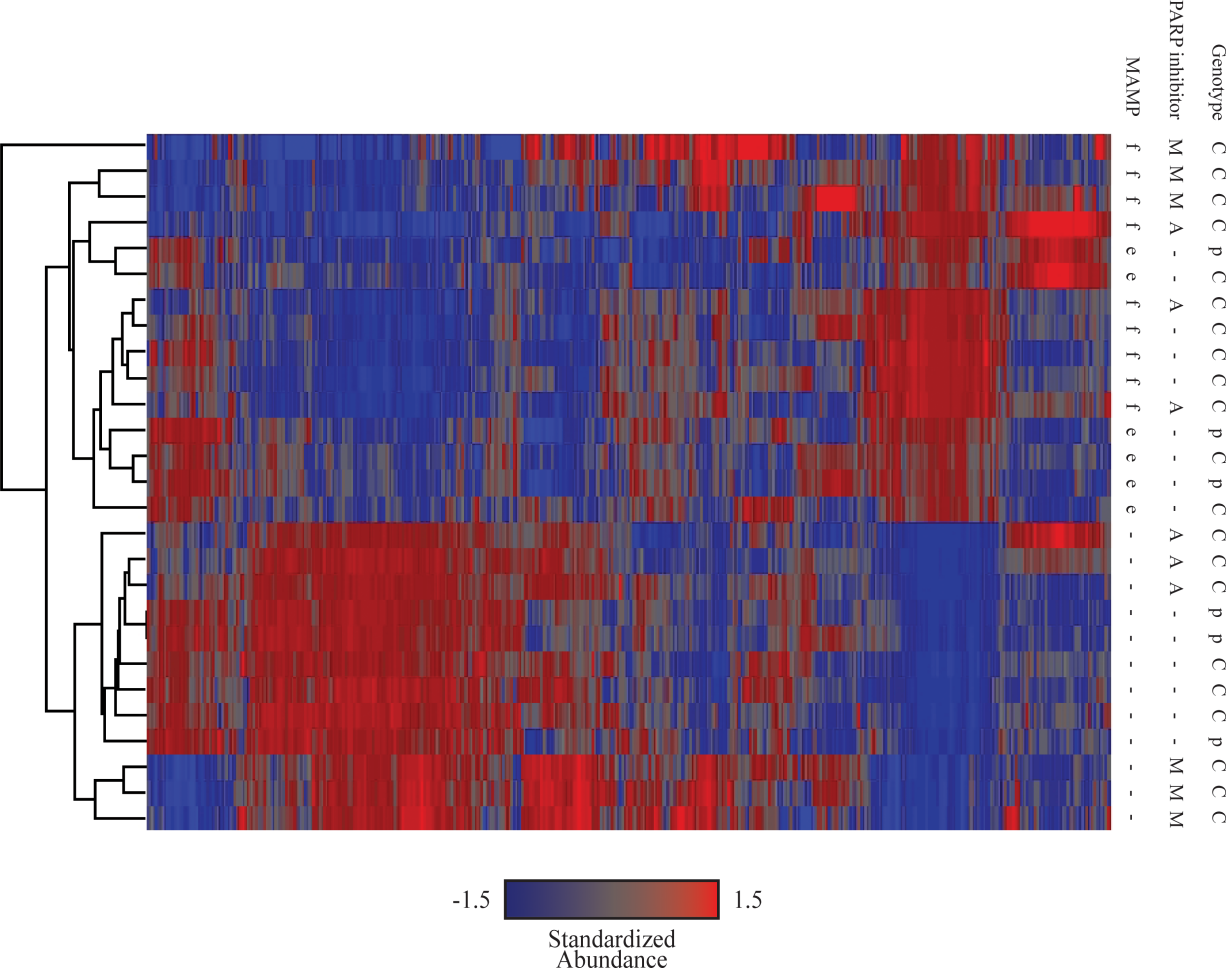
Hierarchical clustering of expression patterns for all 30,387 genes present on 1-plex Nimblegen *Arabidopsis thaliana* array. Standardized transcript abundances (mean = 0, standard deviation = 1) for three biological replicates of nine treatments (Col-0 untreated, Col-0 + flg22, Col-0 + 3AB, Col-0 + 3MB, Col-0 + flg22 + 3AB, Col-0 + flg22 + 3MB, Col-0 + elf18, *parg1-2* untreated, and *parg1-2* + elf18) were used to determine Euclidean distances between treatments and genes, represented by the left and top dendrograms, respectively. f = 1μM flg22, e = 1μM elf18, A = 2.5mM 3-aminobenzmide (3AB), M = 2.5mM 3-methoxybenzamide (3MB), C = Col-0 (wild-type), p = *parg1-2*.

Hierarchical clustering was performed on the entire data set, using the average for technical replicates but keeping biological replicates separate, to allow visual inspection of reproducibility across replicates and to identify relative similarity of overall mRNA abundance profiles after the different treatments (Fig 2). For each gene, standardized transcript abundances were calculated with z scores to achieve mean = 0, standard deviation = 1.

To identify genes exhibiting significant mRNA abundance differences between treatments, relative mRNA abundances for each gene were then calculated from the average of the technical replicates (2) and biological replicates (3) for a total of six replicates per treatment (S5 Table). These values were log_2_ transformed, and further analysis of the data was carried out using Partek Genomics Suite v6.10 (Partek, St. Louis, MO, USA). General analysis of variance (ANOVA) tests of significance were used that give more discriminating q-values for false-discovery rate (FDR) (Benjamini Hochberg) than the typical p-values or q-values captured by many widely-used analysis platforms. Principal components analysis (PCA) revealed mean F ratios for batch (replicate), label (treatment/genotype), and error were 1.71, 17.20, and 1.00, respectively.

**Fig 3:**
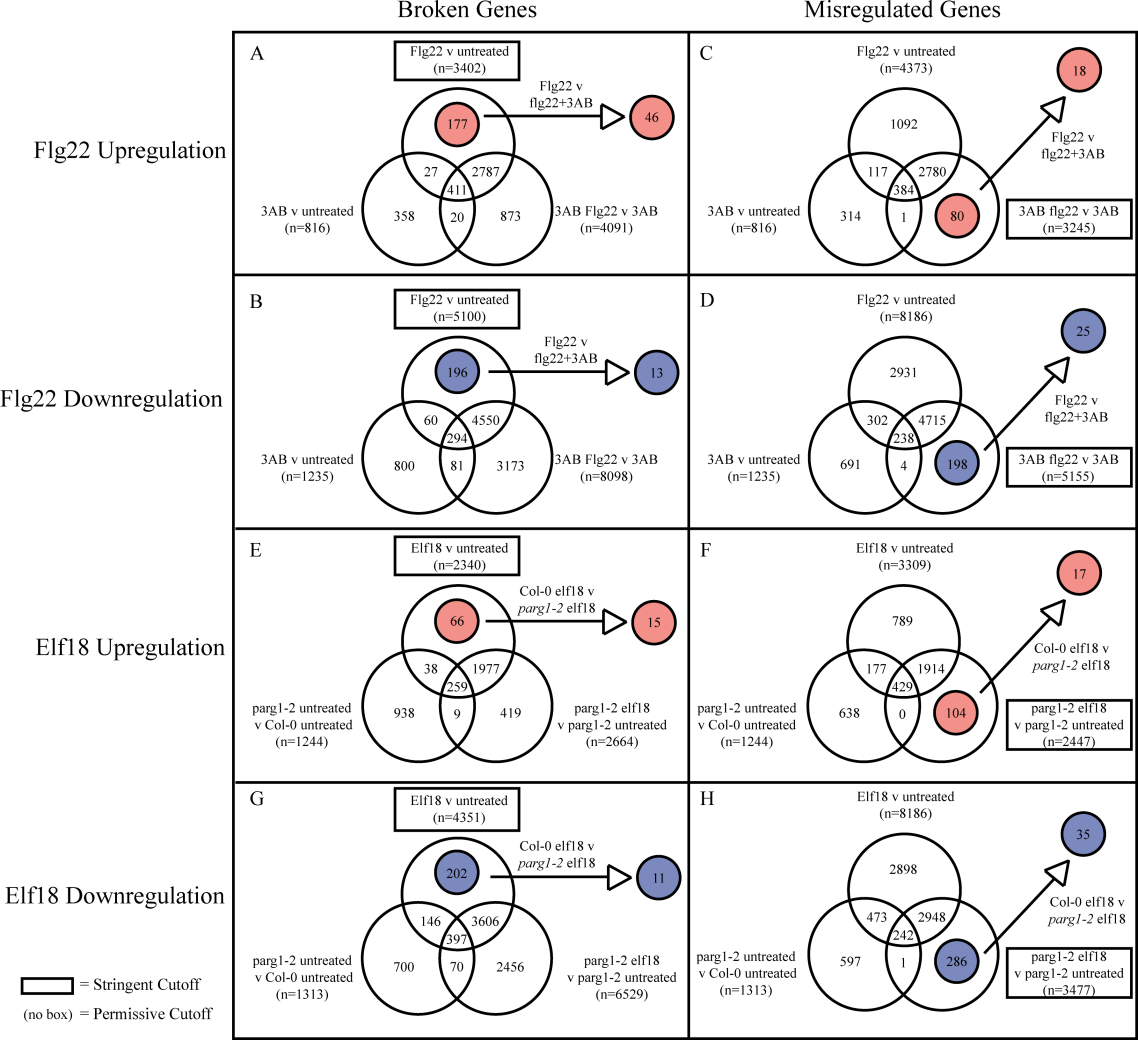
Determination of differentially regulated genes. After initial analysis (see methods), lists of genes differentially regulated between flg22 and flg22 + 3AB (**A-D**) or between Col-0 elf18 and *parg1-2* elf18 (**E-H**) were assembled. Upregulated (red) (**A, C, E, F**) and downregulated (blue) (**B, D, G, H**) genes were determined separately. “Broken” genes were defined for this study as those genes that displayed statistically significant differences in mRNA abundance after treatment with MAMP (flg22 or elf18) (stringent cutoff = FDR <0.05, fold-change>1.3 or <-1.3) versus untreated, but for which MAMP treatment in the presence of 3AB or *parg1-2* did not cause a statistically significant difference (even at the relatively permissive cutoff of p<0.05, fold-change>1.0 or <-1.0). “Misregulated” genes were defined as those genes upon which MAMPs had no statistically significant impact on mRNA abundance in wild-type plants, even using non-stringent cutoff values (p<0.05 and fold-change>1.0 or <-1.0), but for which MAMP treatment did cause significant differences (at stringent cutoff values) in the presence of either 3AB or *parg1-2*. To further reduce the occurrence of Type I false positives, the highlighted gene lists were then filtered once more for flg22 v flg22 + 3AB (p<0.05, fold-change>1.3 or <-1.3) or Col-0 elf18 v *parg1-2* elf18 (p<0.05, fold-change>1.3 or <-1.3).

To identify genes differentially regulated by MAMP treatment compared with no treatment, whose mRNA abundances after MAMP treatment were then altered by 3AB treatment or *parg1-2* mutation, a number of criteria were applied to the entire data set (Fig 3). For example, for Col-0 flg22 +/- 3AB, a large portion of flg22-regulated genes remain flg22-regulated in the presence of 3AB (i.e., 3AB + flg22 compared to 3AB) or their mRNA abundance was also disrupted by 3AB treatment alone compared to untreated controls (and therefore the differential regulation could not be attributed to MAMP-elicitation). Accordingly, of the genes differentially regulated in Col-0 by flg22 (false discovery rate (FDR) < 0.05, fold-change > 1.3 or < -1.3) [59–62], those genes also differentially regulated with 3AB treatment alone (p < 0.05, fold-change > 1.0 or < -1.0) were subtracted from further analysis (see Fig 3 legend for additional rationale). To diminish Type I errors, the remaining genes were then screened for those that exhibited a significant change in mRNA abundance when comparing flg22 versus 3AB + flg22 (by filtering to remove genes that had p > 0.05 or fold changes < 1.3 and > -1.3 for flg22 versus 3AB + flg22) (Fig 3). Gene name, annotation, and expression data for each gene in the final gene lists are presented in S2 Table.

A similar subtraction and filtering was carried out for the *parg1-2* experiment (Fig 3). From the genes differentially regulated between Col-0 untreated and elf18 (FDR < 0.05, fold-change > 1.3 or < -1.3), those genes also differentially regulated by *parg1-2* untreated versus Col-0 untreated (p < 0.05, fold-change > 1.0 or < -1.0), and/or by *parg1-2* + elf 18 versus *parg1-2* untreated (p < 0.05, fold-change > 1.0 or < -1.0), were subtracted out. A filter for Col-0 + elf18 versus *parg1-2* + elf18 (p < 0.05, fold-change > 1.3 or < -1.3) was then applied to identify genes whose mRNA abundance after MAMP treatment was significantly altered by the *parg1-2* knockout.

### Hierarchical clustering of significantly regulated genes

**Fig 4:**
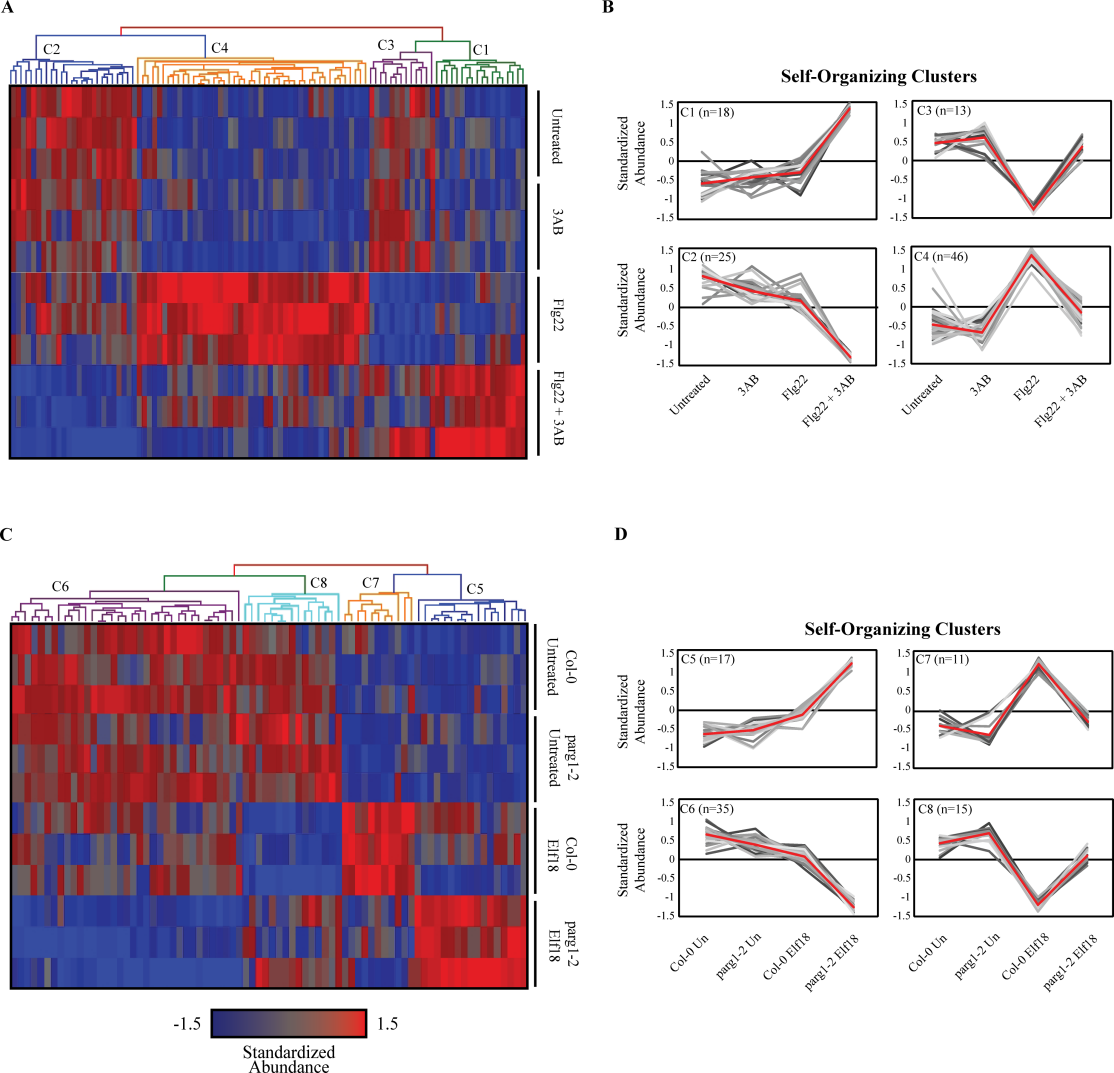
Hierarchical clustering of gene products whose regulation by MAMPs are broken or misregulated by PARP inhibitor or *parg1-2* knockout. **A, C**, Hierarchical clustering of transcript abundance for MAMP-regulated genes identified (see Fig 3) as exhibiting broken or misregulated expression due to 3AB treatment (A, n = 102) or due to *parg1-2* mutation (C, n = 78). Each column represents a single gene and each row a single treatment (all treatments replicated three times); gene abundances standardized to mean = 0, standard deviation = 1.0; red = more abundant, blue = less abundant. Clustering was performed by calculating Euclidean distances on columns, using average linkage scores. **B, D**, Average transcript abundance (y-axis) for each treatment (x-axis), for each gene within the designated color-coded clusters shown at the top of A and C. Red lines denote overall mean mRNA abundance for all genes within the cluster, and grayscale lines represent each individual gene within the cluster.

**Fig 5:**
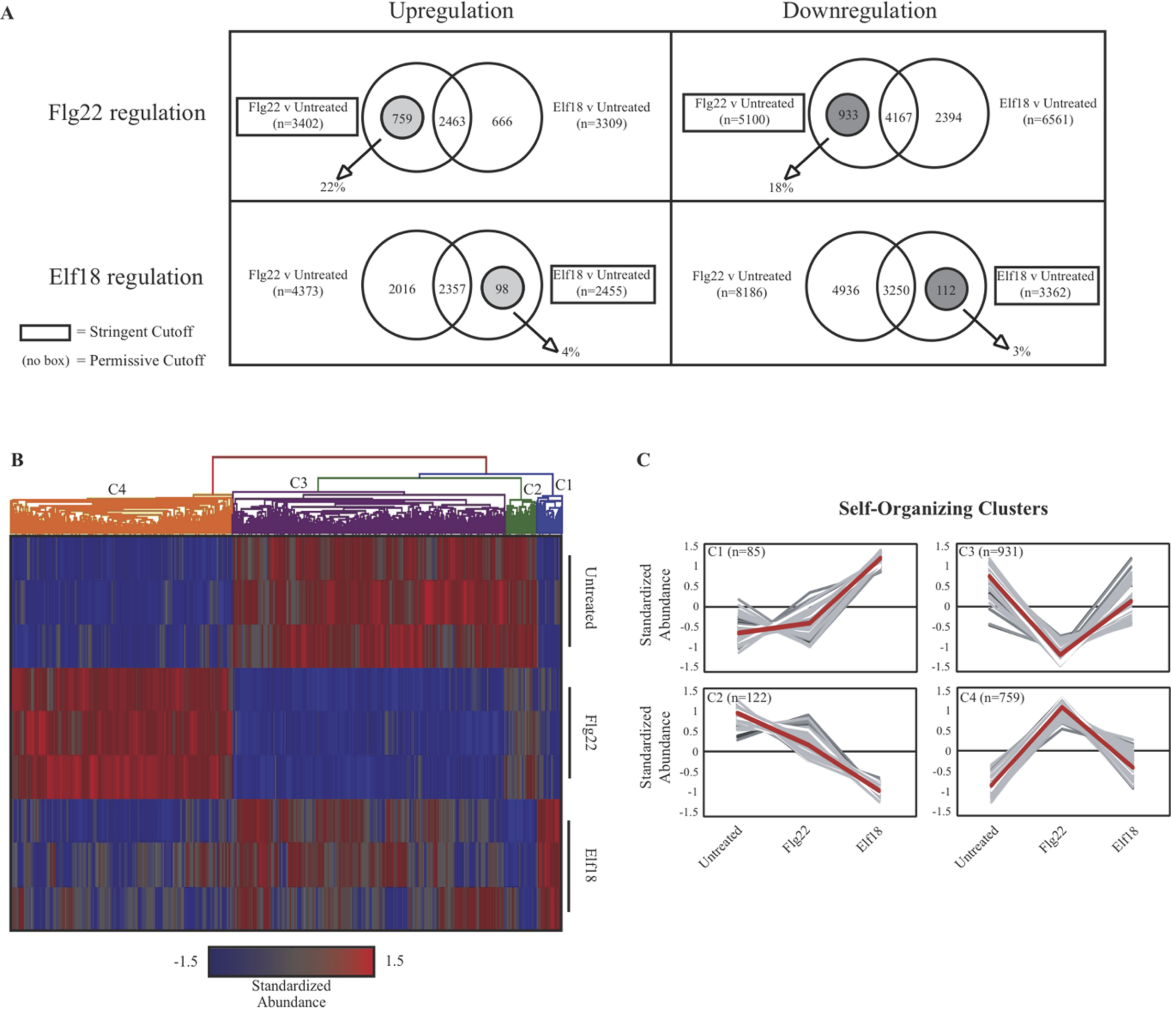
Identification of genes differentially regulated between flg22 and elf18 in wild-type plants. **A**, After initial analysis (see methods), lists of genes differentially regulated between flg22 and elf18 were assembled. Upregulated (red) and downregulated (blue) genes were determined separately. Flg22-regulated genes not differentially regulated by elf18 in these experiments were defined as those that displayed statistically significant differences in mRNA abundance after treatment with flg22 versus untreated (FDR < 0.05, fold-change > 1.3 or < -1.3), which did not display statistically significant difference in mRNA abundance after treatment with elf18 versus untreated even using more permissive cutoff values (p < 0.05, fold-change > 1.0 or <-1.0). The reciprocal calculations were used to determine elf18-regulated genes not differentially regulated by flg22 in these experiments. **B**, Hierarchical clustering of transcript abundance for MAMP-regulated genes identified in A as exhibiting differential regulation by only one of the two studied MAMPs (A, n = 1897). Each column represents a single gene and each row a single treatment (all treatments replicated three times); gene abundances standardized to mean = 0, standard deviation = 1.0; red = more abundant, blue = less abundant. Clustering was performed by calculating Euclidean distances on columns, using average linkage scores. **C**, Average transcript abundance (y-axis) for each treatment (x-axis), for each gene within the designated color-coded clusters shown at the top of A. Red lines denote overall mean mRNA abundance for all genes within the cluster, grayscale lines represent each individual gene within the cluster.

Hierarchical clusters were generated from combined gene lists (Fig 4: 3AB broken genes + 3AB misregulated genes, *parg1*-broken genes + *parg1*-misregulated genes; Fig 5: elf18-regulated genes + flg22-regulated genes). The entire microarray dataset was filtered as described above to obtain the desired lists, and the resulting gene abundances were standardized by calculating z scores (mean = 0, standard deviation = 1). Row and column Euclidean distances were calculated, and row and column clusters were calculated using average linkage. Column clusters were colored based on a user-determined threshold based on visual analysis of the calculated clusters, and these were used to visualize patterns of gene expression across multiple treatments and/or genotypes. For further visualization, average gene intensities were then calculated for each gene for each relevant treatment/genotype within any one cluster.

### Gene ontology enrichment analysis

The GO (gene ontology) analysis software BinGO [63] was used to analyze gene lists for GO term enrichment (Tables 1–4). ANOVA p-values for significantly overrepresented terms were calculated and larger gene lists are required to yield statistically significant output. Therefore, the statistical criteria used for generating the gene lists of Figs 4 and 5 and S1 Table were relaxed. For example, to generate a larger list of flg22-upregulated genes broken by 3AB, the untreated versus flg22 comparison criteria were relaxed from (FDR < 0.05, fold-change > 1.3 or < -1.3) to (FDR < 0.1, fold-change > 1.2 or < -1.2), while the subtraction criteria (such as for 3AB v. untreated and 3AB + flg22 v. 3AB) were relaxed from (p < 0.05, fold-change > 1.0 or < -1.0) to (FDR < 0.1, fold-change > 1.0 or < -1.0). This statistical “loosening” increased this example gene list size from 180 to 538 genes. GO terms that were overrepresented in those gene lists were then identified.

**Table 1:** Gene ontology over-representation in genes whose regulation by flg22 is broken or misregulated by PARP inhibitor.

MAMP	Disruption	GO ID	Description	p-value ^a^	Cluster Frequency ^b^	Total Frequency ^c^
Not regulated by Flg22	Downregulated by 3AB	6342	chromatin silencing	4.34E-02	4/568 0.7%	11/20006 0.0%
Not regulated by Flg22	Downregulated by 3AB	45298	tubulin complex	4.59E-02	4/526 0.7%	12/18595 0.0%
Not regulated by Flg22	Downregulated by 3AB	9535	chloroplast thylakoid membrane	4.59E-02	13/526 2.4%	177/18595 0.9%
Not regulated by Flg22	Downregulated by 3AB	791	euchromatin	4.59E-02	2/526 0.3%	3/18595 0.0%
Not regulated by Flg22	Downregulated by 3AB	30090	photosystem	4.78E-02	4/551 0.7%	26/18595 0.1%
Not regulated by Flg22	Downregulated by 3AB	6629	lipid metabolic process	4.79E-02	28/693 4.0%	509/23786 2.1%
Downregulated by Flg22	Broken by 3AB	5730	nucleolus	2.69E-02	6/551 1.0%	46/18595 0.2%
Downregulated by Flg22	Broken by 3AB	9535	chloroplast thylakoid membrane	2.69E-02	13/551 2.3%	177/18595 0.9%
Downregulated by Flg22	Broken by 3AB	31969	chloroplast membrane	3.02E-02	6/551 1.0%	49/18595 0.2%
Downregulated by Flg22	Broken by 3AB	5840	ribosome	3.02E-02	20/551 3.6%	347/18595 1.8%
Upregulated by Flg22	Broken by 3AB	4872	receptor activity	2.31E-02	11/410 2.6%	181/25179 0.7%
Upregulated by Flg22	Broken by 3AB	4888	transmembrane receptor activity	4.04E-02	10/373 2.6%	153/21036 0.7%
Upregulated by Flg22	Broken by 3AB	4435	phosphoinositide phospholipase C activity	4.04E-02	2/373 0.5%	3/21036 0.0%

^a^Significant Benjamini-Hochberg FDR corrected p-values

^b^Number of genes in differential regulation gene list (see Fig 2) with the specified GO term relative to total number of genes in that gene list for Biological Process, Molecular Function, Cellular Component, or GoSlim Plant terms

^c^Number of GO-annotated genes on array with specified GO term relative to total number of GO-annotated genes represented on array for Biological Process, Molecular Function, Cellular Component, or GoSlim Plant terms

**Table 2:** Gene ontology over-representation in genes whose regulation by elf18 is broken or misregulated by *parg1-2* knockout.

MAMP	Disruption	GO ID	Description	p-value^a^	Cluster Frequency^b^	Total Frequency^c^
Downregulated by Elf18	Broken by *parg1-2*	5840	ribosome	6.03E-05	34/944 3.6%	347/23786 1.4%
Downregulated by Elf18	Broken by *parg1-2*	30873	cytosolic small ribosomal subunit	6.59E-05	10/710 1.4%	44/18595 0.2%
Downregulated by Elf18	Broken by *parg1-2*	5198	structural molecule activity	8.37E-05	39/944 4.1%	440/23786 1.8%
Downregulated by Elf18	Broken by *parg1-2*	3740	structural constituent of ribosome	2.64E-04	34/851 3.9%	328/21036 1.5%
Downregulated by Elf18	Broken by *parg1-2*	6416	translation	3.70E-04	40/944 4.2%	492/23786 2.0%
Downregulated by Elf18	Broken by *parg1-2*	5829	cytosol	5.23E-04	26/944 2.7%	269/23786 1.1%
Downregulated by Elf18	Broken by *parg1-2*	5739	mitochondrion	2.72E-03	53/944 5.6%	806/23786 3.3%
Downregulated by Elf18	Broken by *parg1-2*	6416	translation	2.92E-03	40/788 5.0%	492/20006 2.4%
Downregulated by Elf18	Broken by *parg1-2*	5852	eukaryotic translation initiation factor 3 complex	7.59E-03	3/710 0.4%	6/18595 0.0%
Downregulated by Elf18	Broken by *parg1-2*	6259	DNA metabolic process	1.80E-02	18/944 1.9%	213/23786 0.8%
Downregulated by Elf18	Broken by *parg1-2*	9308	amine metabolic process	2.15E-02	25/788 3.1%	296/20006 1.4%
Downregulated by Elf18	Broken by *parg1-2*	6365	rRNA processing	2.15E-02	6/788 0.7%	25/20006 0.1%
Downregulated by Elf18	Broken by *parg1-2*	6519	amino acid and derivative metabolic process	2.42E-02	26/944 2.7%	368/23786 1.5%
Downregulated by Elf18	Broken by *parg1-2*	5849	mRNA cleavage factor complex	2.62E-02	2/710 0.2%	3/18595 0.0%
Downregulated by Elf18	Broken by *parg1-2*	16801	hydrolase activity, acting on ether bonds	2.64E-02	4/851 0.4%	8/21036 0.0%
Downregulated by Elf18	Broken by *parg1-2*	16043	cellular component organization and biogenesis	2.77E-02	48/944 5.0%	814/23786 3.4%
Downregulated by Elf18	Broken by *parg1-2*	5634	nucleus	2.80E-02	79/944 8.3%	1482/23786 6.2%
Downregulated by Elf18	Broken by *parg1-2*	5744	mitochondrial inner membrane presequence translocase complex	5/11/10	4/710 0.5%	19/18595 0.1%
Downregulated by Elf18	Broken by *parg1-2*	5741	mitochondrial outer membrane	3.45E-02	4/710 0.5%	20/18595 0.1%
Downregulated by Elf18	Broken by *parg1-2*	5730	nucleolus	4.15E-02	6/710 0.8%	46/18595 0.2%
Not regulated by Elf18	Misregulated by *parg1-2*	9790	embryonic development	1.38E-03	26/883 2.9%	277/23786 1.1%
Not regulated by Elf18	Misregulated by *parg1-2*	9793	embryonic development ending in seed dormancy	3.24E-03	25/726 3.4%	256/20006 1.2%
Not regulated by Elf18	Misregulated by *parg1-2*	8026	ATP-dependent helicase activity	3.30E-03	11/726 1.5%	76/21036 0.3%
Not regulated by Elf18	Misregulated by *parg1-2*	9536	plastid	5.58E-03	102/883 11.5%	1911/23786 8.0%
Not regulated by Elf18	Misregulated by *parg1-2*	9507	chloroplast	7.87E-03	94/670 14.0%	1808/18595 9.7%
Not regulated by Elf18	Misregulated by *parg1-2*	9508	plastid chromosome	1.39E-02	4/670 0.5%	11/18595 0.0%
Not regulated by Elf18	Misregulated by *parg1-2*	22804	active transmembrane transporter activity	1.86E-02	29/726 3.9%	425/21036 2.0%
Not regulated by Elf18	Misregulated by *parg1-2*	6506	GPI anchor biosynthetic process	2.57E-02	4/726 0.5%	9/20006 0.0%
Not regulated by Elf18	Misregulated by *parg1-2*	16123	xanthophyll biosynthetic process	3.60E-02	3/726 0.4%	5/20006 0.0%
Not regulated by Elf18	Misregulated by *parg1-2*	30127	COPII vesicle coat	4.73E-02	3/670 0.4%	9/18595 0.0%
Upregulated by Elf18	Broken by *parg1-2*	16740	transferase activity	2.91E-02	43/281 15.3%	2168/23786 9.1%
Upregulated by Elf18	Broken by *parg1-2*	51707	response to other organism	2.95E-02	12/224 5.3%	284/20006 1.4%
Upregulated by Elf18	Broken by *parg1-2*	50665	hydrogen peroxide biosynthetic process	2.95E-02	2/224 0.8%	2/20006 0.0%
Upregulated by Elf18	Broken by *parg1-2*	45087	innate immune response	3.52E-02	7/224 3.1%	115/20006 0.5%
Upregulated by Elf18	Broken by *parg1-2*	9268	response to pH	3.52E-02	2/224 0.8%	3/20006 0.0%

^a^significant Benjamini-Hochberg FDR corrected p-values

^b^number of genes in differential regulation gene list (see Fig 2) with the specified GO term relative to total number of genes in that gene list for Biological Process, Molecular Function, Cellular Component, or GoSlim Plant terms

^c^number of GO-annotated genes on array with specified GO term relative to total number of GO-annotated genes represented on array for Biological Process, Molecular Function, Cellular Component, or GoSlim Plant terms

**Table 3:** Elf18-regulated genes broken by *parg1-2* knockout that are involved in innate immunity.

Enzyme/Protein	Gene Abbreviation	Gene ID
FMN-linked oxidoreductase superfamily protein	-	AT1G17990
TGA1A-Related Gene 3	*TGA3*	AT1G22070
Ethylene Response 1	*ETR1*	AT1G66340
HOPW1-1-Induced Gene1	*HWI1*	AT1G70690
Pathogenesis-Related Gene 5	*PR5*	AT1G75040
Cyclic Nucleotide-Gated Channel 12	*ATCNGC12*	AT2G46450
Recognition of Peronospora Parasitica 13	*RPP13*	AT3G46530
Avirulence-Responsive Family Protein, AIG1 Family Protein	-	AT4G09930
UDP-Glucosyltransferase 73B2	*UGT73B2*	AT4G34135
Anthranilate Synthase Alpha Subunit 1	*ASA1*	AT5G05730
AvrPphB Susceptible 1	*PBS1*	AT5G13160
Dicer-Like 4	*DCL4*	AT5G20320

**Table 4:** Gene ontology over-representation in genes differentially regulated by either flg22 or elf18.

MAMP Regulation	GO ID	Description	p-value^a^	Cluster Frequency^b^	Total Frequency^c^
Downregulated by Flg22	9733	response to auxin stimulus	9.70E-07	26/692 3.7%	204/20916 0.9%
Downregulated by Flg22	45449	regulation of transcription	2.46E-04	68/692 9.8%	1139/20916 5.4%
Downregulated by Flg22	9738	abscisic acid mediated signaling	2.73E-02	4/692 0.5%	13/20916 0.0%
Downregulated by Flg22	3700	transcription factor activity	2.47E-05	72/700 10.2%	1207/21958 5.4%
Downregulated by Flg22	47209	coniferyl-alcohol glucosyltransferase activity	2.62E-03	3/700 0.4%	3/21958 0.0%
Downregulated by Flg22	5634	nucleus	1.33E-02	74/606 12.2%	1510/19300 7.8%
Downregulated by Flg22	786	nucleosome	3.68E-02	7/606 1.1%	47/19300 0.2%
Downregulated by Flg22	323	lytic vacuole	4.84E-02	3/606 0.4%	8/19300 0.0%
Downregulated by Flg22	9719	response to endogenous stimulus	3.86E-08	45/801 5.6%	499/25179 1.9%
Downregulated by Flg22	30528	transcription regulator activity	2.79E-06	84/801 10.4%	1441/25179 5.7%
Downregulated by Flg22	5634	nucleus	2.78E-03	74/801 9.2%	1510/25179 5.9%
Downregulated by Flg22	9628	response to abiotic stimulus	1.74E-02	34/801 4.2%	607/25179 2.4%
Upregulated by Flg22	6952	defense response	4.30E-02	28/539 5.1%	499/20916 2.3%
Upregulated by Flg22	5992	trehalose biosynthetic process	4.30E-02	4/539 0.7%	13/20916 0.0%
Upregulated by Flg22	6468	protein amino acid phosphorylation	4.30E-02	37/539 6.8%	798/20916 3.8%
Upregulated by Flg22	30001	metal ion transport	4.30E-02	11/539 2.0%	130/20916 0.6%
Upregulated by Flg22	6355	regulation of transcription, DNA-dependent	4.30E-02	28/539 5.1%	577/20916 2.7%
Upregulated by Flg22	4888	transmembrane receptor activity	8.02E-04	16/575 2.7%	153/21958 0.6%
Upregulated by Flg22	3700	transcription factor activity	9.00E-04	58/575 10.0%	1207/21958 5.4%
Upregulated by Flg22	4805	trehalose-phosphatase activity	4.98E-03	4/575 0.6%	10/21958 0.0%
Upregulated by Flg22	46872	metal ion binding	1.72E-02	54/575 9.3%	1296/21958 5.9%
Upregulated by Flg22	16298	lipase activity	3.37E-02	10/575 1.7%	122/21958 0.5%
Upregulated by Flg22	17076	purine nucleotide binding	3.37E-02	35/575 6.0%	781/21958 3.5%
Downregulated by Elf18	8026	ATP-dependent helicase activity	9.11E-03	4/93 4.3%	76/21958 0.3%
Downregulated by Elf18	4152	dihydroorotate dehydrogenase activity	4.93E-02	1/93 1.0%	1/21958 0.0%
Downregulated by Elf18	9905	ent-copalyl diphosphate synthase activity	4.93E-02	1/93 1.0%	1/21958 0.0%
Downregulated by Elf18	8676	3-deoxy-8-phosphooctulonate synthase activity	4.93E-02	1/93 1.0%	1/21958 0.0%
Upregulated by Elf18	9733	response to auxin stimulus	2.47E-04	7/68 10.2%	204/20916 0.9%
Upregulated by Elf18	46686	response to cadmium ion	1.69E-03	3/68 4.4%	21/20916 0.1%
Upregulated by Elf18	9651	response to salt stress	1.66E-02	4/68 5.8%	135/20916 0.6%
Upregulated by Elf18	9753	response to jasmonic acid stimulus	1.66E-02	3/68 4.4%	61/20916 0.2%
Upregulated by Elf18	9751	response to salicylic acid stimulus	2.07E-02	3/68 4.4%	68/20916 0.3%

^a^Significant Benjamini-Hochberg FDR corrected p-values

^b^Number of genes in differential regulation gene list (see Fig 4) with the specified GO term relative to total number of genes in that gene list for Biological Process, Molecular Function, Cellular Component, or GoSlim Plant terms

^c^Number of GO-annotated genes on array with specified GO term relative to total number of GO-annotated genes represented on array for Biological Process, Molecular Function, Cellular Component, or GoSlim Plant terms

### Knockout lines

Homozygous SALK T-DNA knockout lines for each of the selected genes were identified as described [64] (ABRC, Columbus, OH, USA). The *parg1-2* mutant allele corresponds to SALK_116088; all other knockout lines are shown in S4 Table. Those knockout lines that showed altered MAMP-response phenotypes were then confirmed by RT-PCR to exhibit absent or reduced mRNA abundance for the designated knockout gene.

### Seedling growth inhibition assays

Flg22-induced seedling growth inhibition assays were performed as described [4,65]. Briefly, twelve Arabidopsis seedlings per treatment were grown on 0.5× Murashige-Skoog (MS) agar media supplemented with 1.5% (w/v) sucrose and 1× Gamborg’s vitamins for 5 days and then transferred to 24-well plates (1 seedling per well) containing 400 μL of liquid 0.5×MS salts, 1.5% (w/v) sucrose, and 1× Gamborg’s vitamins media. Seedlings were then treated with a final concentration of 0.05 μM (low) or 1.0 μM (high) flg22, and fresh weight was recorded 14 days later.

### Callose deposition

Twelve Arabidopsis seedlings per treatment were grown on 0.5× MS, 1.5% (w/v) sucrose, and 1× Gamborg’s vitamins media for five days and then transferred to 24-well plates (one seedling per well) containing 400 μL of liquid 0.5× MS salts, 1.5% (w/v) sucrose, and 1× Gamborg’s vitamins media. Seedlings were then treated with a final concentration of 1.0 μM flg22. After an additional 24h, seedlings were fixed overnight in formaldehyde/acetic acid/ethanol (FAA), cleared with 100% ethanol, and stained in 0.01% aniline blue (67mM K_2_HPO_4_ pH12). Callose was visualized using ultraviolet epifluorescence microscopy [4]. Independent experiments were performed three times with similar results. In high-throughput screens, callose deposits were qualitatively scored using a scale of zero to five: 0, no callose deposits; 1, single isolated callose deposits across entire cotyledon; 2, very few deposits scattered across entire cotyledon; 3, moderate coverage of callose deposits across otherwise unstained regions; 4, many callose deposits covering entire surface of cotyledon but with some unstained space remaining; 5, uniform callose deposits covering entire surface of cotyledon. Callose deposits were also quantified by calculating area of callose deposits based on the difference in hue compared with the background hue of each leaf (ImageJ, NIH, Rockville, MD).

### RNA extraction and gene expression analysis

Total RNA was extracted from eight-day-old whole seedlings grown in 500 μl MS media + 1% sucrose and treated with 1 μM flg22 peptide (Qiagen, Valencia, CA, USA). Contaminating DNA was removed using a DNA-free DNase Treatment and Removal Kit (Thermo Scientific, Rockford, IL, USA), and RNA concentrations were quantified by Nanodrop Spectrophotometer (Thermo Scientific, Rockford, IL, USA). Semi-quantitative RT-PCR reactions were confirmed to be using a non-saturating number of PCR cycles; reactions contained cDNA (synthesized with M-MLV Reverse Transcriptase and Oligo-dT priming) (Promega, Madison, WI, USA), and corresponding gene-specific primers pairs (IDT, Coralville, IA, USA):

5′-ATGGACGAAGGAGACCTAG-3′ and 5′-CTTTTCTTTGATTTGGATTCTG-3′ (*WRKY29*); 5’-TACTATTCGACTCGCCAAATG-3’ and 5’-CTACCTTGCTCGAGGAACC-3’ (*FRK1*); 5′-AGGTTCTGTTCCAGCCATC-3′ and 5′-TTAGAAGCATTTCCTGTGAAC-3′ (*Actin-2*); 5’-CTCATGCTCAGTATGATGC-3’ and 5’CTCCAATCTTCTCGTCTATC-3’ (*CYP81F2*); 5’-ACAAATGGTCTGCTATAGCT-3’ and 5’-CTTGTGTGTAACTGGATCAA-3’ (*MYB51*).

### Data deposition

Raw data and normalized calls from the microarray presented here have been deposited in GEO (http://www.ncbi.nlm.nih.gov/geo)), #GSE100205. A summary table with abundance values for each treatment replicate for all 60,770 gene probes, as well as average normalized call values and fold-change versus untreated Col-0, is also available as an Excel file (S5 Table).

## Results

### Expression profiling of plant response to MAMPs when poly(ADP-ribosyl)ation is altered

An expression profiling experiment was designed primarily to characterize two phenomena: first, the effect of PARP inhibition on the transcriptional response to flg22; and second, the effect of knocking out *PARG1* expression on the transcriptional response to elf18 (Fig 1A and 1B). Because the treatments for both experiments were carried out simultaneously for any given biological replicate, additional comparisons could be made to assess two additional comparisons: first, the differences between basal mRNA levels (without MAMP treatment) between wild-type, *parg1-2* knockouts, and plants treated with PARP inhibitors; and second, the differences between wild-type Arabidopsis responses to flg22 and elf18.

The experimental design included three replicates of nine genotype/treatment conditions (Col-0 treated with 3AB, 3MB, flg22, elf18, 3AB + flg22, or 3MB + flg22, or untreated; and *parg1-2* untreated or treated with elf18) for a total of 27 samples. Principal component analysis was used to identify sources of variation in the data set; mean F ratios for batch (replicate), label (treatment/genotype), and error were 1.71, 17.20, and 1.00, respectively, indicating that batch contributed very little to variation, while genotype and treatment were responsible for most of the variation present. These F ratios indicate high reproducibility between biological replicates.

To confirm proper basal defense elicitation by the MAMP peptides used, patterns of defense gene expression in wild-type plants were examined. Within 90 minutes of MAMP treatment, Arabidopsis *WRKY DNA Binding Protein 29 (WRKY29)* (At4g23550), *Flg22-Induced Receptor-like Kinase 1 (FRK1)* (At2g19190), *MYB Domain Protein 51 (MYB51)* (At1g18570) and *Cytochorome P450*, *Family 81*, *Subfamily F*, *Polypeptide 2 (CYP81f2)* (At5g57220) genes are upregulated [7,66], and *TIR1* (At3g62980) gene expression is downregulated [67]. This pattern of gene expression was confirmed in our study in the Col-0 flg22-treated samples, compared to untreated plants (S6 Table).

### Impact of PARP inhibitors and *parg1* knockout on untreated plants

The effect of PARP inhibitor treatment and *PARG1* knockout on otherwise untreated or wild-type plants was first investigated. When all 30,387 predicted genes represented on the arrays were hierarchically clustered, 3AB treatment clustered closely with untreated plants, indicating very few pleiotropic effects (Fig 2). Only 228 genes displayed statistically significant alterations in mRNA abundances after 3AB treatment (contrast, for example, to flg22 treatment, in which 3402 genes displayed a significant difference in abundance using the same criteria). This result is consistent with earlier observations that 3AB-treated plants phenotypically resemble untreated plants [12], and that 3AB treatment does not induce visually evident generalized stress in plants in the absence of other stressors. In contrast, 3MB treatment elicited significant differences in the gene expression of 3935 genes relative to untreated plants. These data indicate that 3MB substantially impacts the physiological status of otherwise untreated plants grown in favorable conditions, and so subsequent analyses for PARP inhibition focused on data acquired using 3AB rather than 3MB. *parg1-2* and wild-type Col-0 untreated plants also clustered closely in the above hierarchical cluster, with only 128 genes showing altered mRNA abundance between untreated Col-0 and *parg1* plants, again indicating few pleiotropic effects in low-stress conditions (Fig 2).

### Transcription profile of flg22 response in PARP inhibitor-treated plants

Within one hour of exposure, flg22 induces a wide range of transcriptional responses in Arabidopsis [67,68]. It was previously observed that 3AB treatment did not block flg22-induced ROS production or elevated expression of the signature flg22-induced genes *WRKY29* and *FRK1*, but did disrupt a number of downstream events in the plant response to flg22 [7]. A primary purpose of the present study was to identify genes whose mRNA abundances are differentially regulated by flg22, but that are no longer differentially regulated by flg22 in the presence of 3AB (referred to here as “broken” genes), as well as those genes up or downregulated by flg22 only in the presence of 3AB (referred to here as “misregulated” genes). The specific tests used for gene selection, and the number of genes in each sub-group, are presented in Fig 3A–3D, and the complete gene lists are provided in S2 Table.

The 102 genes present in the final gene lists generated as described above were then visualized by hierarchical clustering (Fig 4A). They grouped in four self-organizing clusters C1-C4 (Fig 4B). C1 and C2 contain those genes normally not under flg22 regulation that are either flg22 up- or downregulated, respectively, in the presence of 3AB (3AB-misregulated genes). C3 and C4 contain those gene products up- or downregulated by flg22 that are no longer differentially regulated by flg22 in the presence of 3AB (3AB-broken genes).

### Transcription profile of elf18 response in *parg1* knockout plants

Two independent *parg1* mutant alleles have been previously shown to display a number of defense phenotypes, including hyper-responsiveness to elf18 and greater susceptibility to necrotroph infection [7]. For the present study, experiments were designed to detect early transcriptional events after elf18 treatment that are disrupted or overactivated by loss of *PARG1*. Genes were identified whose mRNA abundances were affected by elf18 in wild-type plants but not in *parg1* mutant plants (*parg1*-broken genes), as well as those regulated by elf18 only in *parg1* knockout plants (*parg1*-misregulated genes). Logic similar to that used for the PARP inhibitor experiments was used (Fig 3), generating a stringent list of those genes whose response to elf18 is broken or misregulated in *parg1-2* plants (S1 Table).

The 78 *parg1-*broken and -misregulated elf18 response genes (Fig 3E and 3H, S2 Table) were then subjected to hierarchical clustering (Fig 4C). They grouped in 4 self-organizing clusters C5-8 (Fig 4D). C5 and C6 contain those genes normally not under elf18 regulation whose mRNA abundances are either elf18 up- or downregulated, respectively, in the *parg1-2* genetic background (*parg1-2* misregulated genes). C7 and C8 contain those genes up or down regulated by elf18, respectively, that are no longer differentially regulated by elf18 in the *parg1-2* plants (*parg1-2* broken genes).

### GO-term analysis of flg22- and elf18-regulated transcriptional responses that are altered by disruption of poly(ADP-ribosyl)ation

To identify specific cellular pathways that may be regulated by poly(ADP-ribosyl)ation during MAMP-elicited immunity, we analyzed the above lists (Fig 3) of broken and misregulated genes for overrepresentation of Gene Ontology (GO) terms. Among genes classified as broken by 3AB, overrepresented GO terms include nucleolus, chloroplast (membrane and thylakoid membrane), receptor activity, and phospholipase C activity. Overrepresented GO terms for 3AB-misregulated genes included chloroplast (photosystem and thylakoid membrane genes), lipid metabolism, and chromatin silencing related genes (Table 2).

Some of the GO terms overrepresented among those genes misregulated during MAMP response in a *parg1-2* mutant background include COPII vesicle coat, GPI anchor biosynthesis, and xanthophyll biosynthesis genes. GO terms that were overrepresented among those genes whose expression was broken during MAMP response in a *parg1-2* mutant background include protein and RNA processing, mitochondrial translocation, and, of particular interest, response to other organisms/innate immune response genes (Table 3). Table 4 lists those genes associated with the GO term “innate immune response” that are normally upregulated by elf18 and are then no longer activated by elf18 in *parg1-2* seedlings. These genes do not fall into a single pathway or branch of any known defense response pathway, but are instead spread across a number of aspects of the innate immune response. Regulation of expression of a number of processes associated with plant defense are altered by the absence of the *PARG1* gene product, including calcium influx (*AtCNGC12*), NPR1-regulated transcription (*TGA3*), trans-acting anti-viral dicer-like activity (*DCL4*), recognition of pathogen effector molecules (*PBS1*), defense-activated tryptophan and chorismate production (*ASA1*), oxidative stress-induced flavonoid glycosylation (*UGT73B2*), the defense gene *PR5*, and the R gene *RPP13*.

### The MAMPs flg22 and elf18 elicit separate, yet overlapping transcriptional responses

The hypothesis that flg22 and elf18 elicit the same set of basal defense responses was also examined. To identify genes in our experiment that were regulated only by flg22 or elf18 but not both, a modified version of the Venn diagram method described above (see Fig 3 legend) was used (Fig 5A), and genes up or downregulated only by on or the other MAMP were identified (S3 Table). These gene lists were then combined to perform hierarchical clustering (Fig 5B) and self-organizing cluster analysis (Fig 5C). It is noteworthy that elf18 regulated most of the same genes as flg22, but flg22 elicited a set of additional responses above and beyond those shared by both MAMPs. Approximately 2000 genes showed altered mRNA abundances with only one of the two studied MAMPs. Up to 20% of flg22-regulated gene products were not regulated by elf18, whereas only 3–4% of elf18-regulated gene products were not also regulated flg22.

Table 5 outlines the overrepresented GO terms present in these clusters of gene products regulated by only one of the two MAMPs studied. Of particular interest are a number of defense genes only induced one hour after treatment by flg22 and not by elf18. These genes include a number of TIR-NBS-LRR class genes, as well as *PAL1*, *Respiratory Burst Oxidase Homolog A (RBOHA)*, *WRKY70* and *CDPK-Related Kinase 4 (CRK4)*. In addition, a number of auxin-responsive gene products showed reduced abundance only after flg22 treatment, as well as other auxin-responsive genes upregulated only by elf18.

**Table 5:** Summary of seedling growth inhibition and callose deposition assays for candidate genes giving a differential response in at least one knockout plant line compared with wild-type Col-0 plants.

Gene number	Gene name	TDNA line	Insertion site(s)	Knockout plant	Callose phenotype^a^	SGI phenotype^b^
At1g47370	Response to bacterial type III effector protein Hopba1 (*RBA1*), TIR-only receptor	SALK_024124C	intron	*rba1-1*	WT	WT
	TIR-only receptor	SALK_037091C	exon	*rba-2*	-	WT
At3g10160	Folylpolyglutamate synthetase 2 (*FPGS2*)	SAIL_510_E12	exon	*fpgs2-2*	WT	-
		SALK_008883C	exon, promoter of At3g10150	*fpgs2-1*	WT	WT
At3g55630	Folylpolyglutamate synthetase 3 (*FPGS3*)	SALK_038762C	promoter, 5'UTR, 3'UTR of 3g55620	*fpgs3-1*	WT/+	-
		SALK_116244C	promoter, 5'UTR	*fpgs3-2*	+	WT
At5g05980	Folylpolyglutamate synthetase 1 (*FPGS1*)	SALK_032544C	promoter, 5'UTR, exon	*fpgs1-2*	+	-
		SALK_015472C	exon	*fpgs1-1*	+	WT
At5g15660	F-box and associated interaction domains-containing protein	SALK_036563C	exon	*f-box-1*	-	WT
		SALK_047400C	5'UTR	*f-box-2*	WT	WT
At5g64640	Plant invertase/pectin methylesterase inhibitor superfamily	SALK_063593C	intron	*pmei-2*	-	-
		SALK_121275C	exon	*pmei-1*	WT	WT

^a^(-) less or (+) more percent area than Col-0 treated with 1uM flg22

^b^(-) larger fresh weight (less seedling growth inhibition) than Col-0 treated with 1uM flg22 for 10 days

### Gene knockout studies for candidate genes

**Fig 6:**
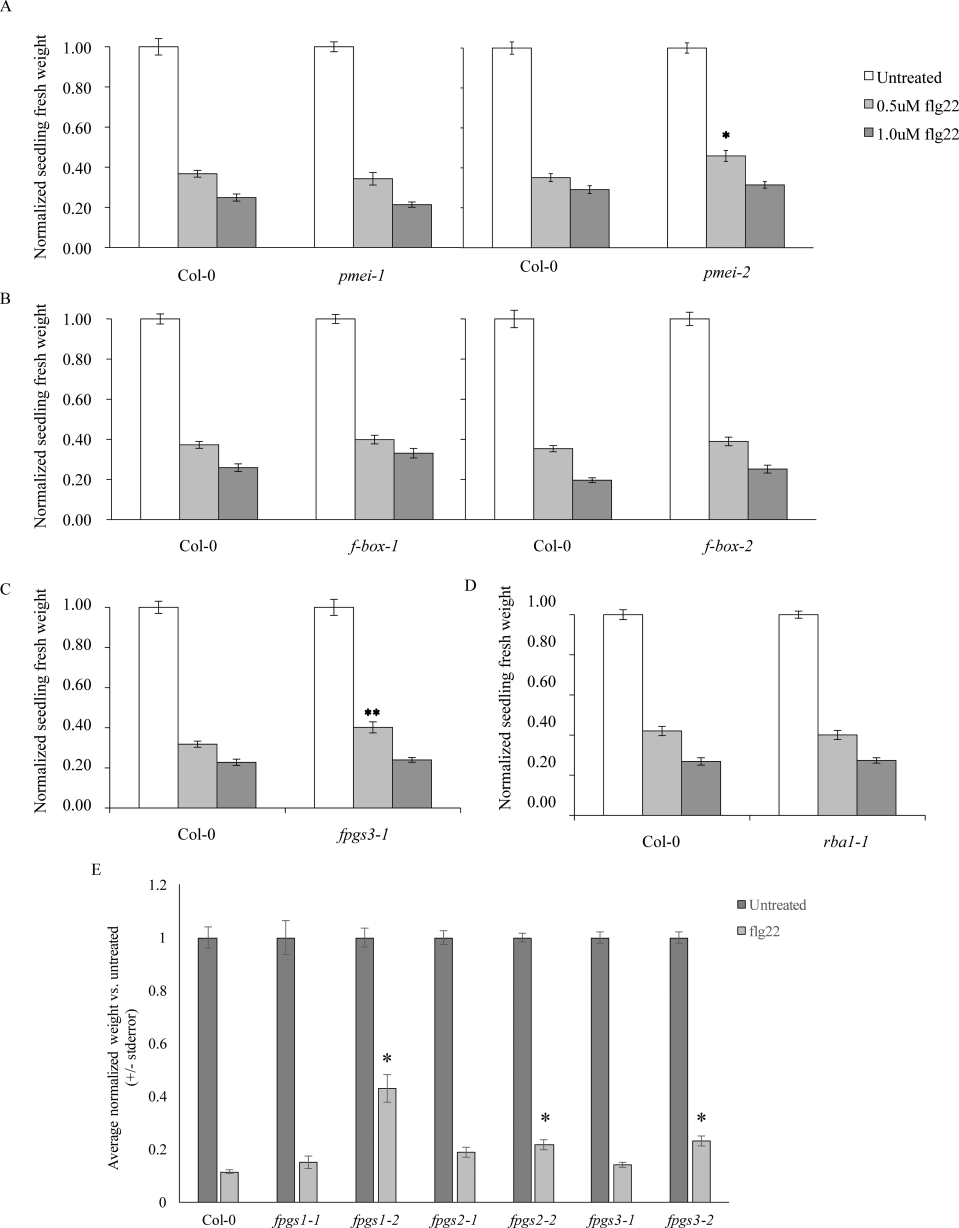
Seedling growth inhibition assay. Five-day-old seedlings of the indicated genotypes were treated with 0.05uM (low) or 1.0uM (high) flg22 peptide and grown for an additional 14 d. Fresh seedlings weights were then recorded and normalized to the average untreated weight within each genotype. A. Pectin methylesterase inhibitor (*PMEI*) (At5g64640) knockouts versus untreated, three (*pmei-1*) and four (*pmei-2*) biological replicates of 12 seedlings per treatment. B. F-box domain-containing gene (At5g15660) knockouts versus untreated, three (*f-box-1*) and two (*f-box-2*) biological replicates of 12 seedlings each. C. *Folylpolygutamate synthetase 3 (FPGS3)* (At3g55630) knockout versus untreated, three biological replicates. D. TIR-X domain-containing gene (At1g47370), three biological replicates. Asterisks summarize ANOVA results across all experiments for tests of similarity of means between the mutant genotype and wild-type plants treated with the same concentration of flg22. E. *FPGS1* (At5g05980), *FPGS2* (At3g10160), and *FPGS3* (At3g55630) versus untreated, three biological replicates. (Tukey's simultaneous test: * *P* < 0.05; **P < 0.005; no asterisk, *P* > 0.05).

**Fig 7:**
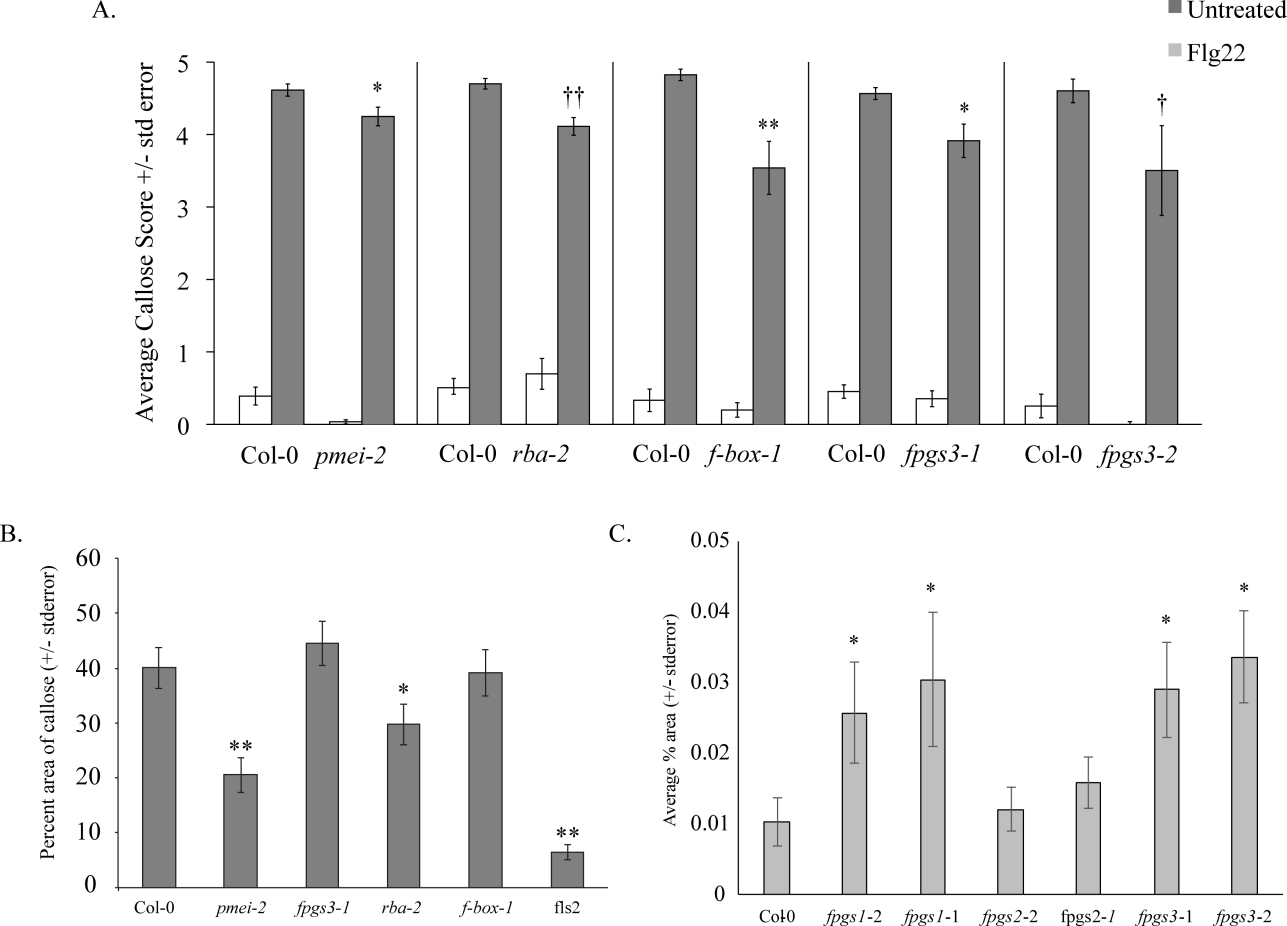
Callose deposition assay. A. 10-day-old Arabidopsis seedlings were treated with distilled, deionized water (H_2_O) or 1 μm flg22, fixed 24 h after flg22 elicitation, and visualized for callose deposition by aniline blue staining and epifluorescence microscopy. Degree of callose deposition was categorized using a scale of 0 to 5, 0 = no callose deposits, 5 = dense callose deposits over entire field of view. Twelve cotyledons per genotype were examined and compared to wild-type (Col-0) responses per biological replicate (n). *pmei-2* (At5g64640) n = 1; *rba-2* (At1g47370) n = 5; *f-box-1* (At5g15660) n = 4; *fpgs3-1* and *fpgs3-2* (At3g55630) n = 4, n = 1, respectively. Asterisks summarize ANOVA results across all experiments for tests of similarity of means between the mutant genotype and wild-type plants treated with flg22 (Tukey's simultaneous test: †P<0.15; ††P<0.1; *P<0.05; **P<0.005; no asterisk, P > 0.05). B. For selected lines, callose deposits were quantified as average percent area covered by white pixels within the viewfield, corresponding to flg22-induced callose, +/- standard error. Ten cotyledons per genotype were examined for each of four biological replicates. Asterisks summarize ANOVA results across all experiments for tests of similarity of means between the mutant genotype and wild-type plants treated with flg22 (Tukey's simultaneous test: *P < 0.055; **P < 0.005; no asterisk, P > 0.05). Representative flg22-treated cotyledons for each genotype in B are shown in C.

Disrupting poly(ADP-ribosyl)ation mechanisms disrupts a subset of plant innate immune responses [7,12], including callose deposition and seedling growth inhibition. We therefore hypothesized that knockouts of some of the genes identified in the above transcriptional profiles (ie., those genes downstream of PARP or PARG activation) will alter some plant defense responses. Homozygous Arabidopsis T-DNA knockout plant lines for genes selected from a subset of the categories in Figs 3 and 4, the gene products whose regulation by MAMP is then broken or misregulated by PARP inhibitor treatment or *parg1-2* knockout. Of the 178 genes represented by lists C1-C4, 54 homozygous T-DNA knockouts were available and initially screened (S4 Table). We did not investigate genes with previously confirmed roles in plant defense. Plants were first screened for altered MAMP-induced seedling growth inhibition and callose deposition, two downstream indicators of detection and response to MAMPs. Of the 54 plant lines screened, one (At3g55630) showed attenuated seedling growth inhibition and 3 (At1g47370, At3g55630, and At5g15660) displayed reduced callose deposition phenotypes, including one knockout (At3g55630) with both seedling growth inhibition (Fig 6) and callose phenotypes (Fig 7). Table 1 provides a summary of these experiments, including tests with second knockout alleles that are discussed below.

### A folylpolyglutamate synthetase is required for wild-type responses to MAMPs

At3g55630 was identified from the microarray as a *parg1-2* misregulated gene whose expression is only downregulated by elf18 in *parg1* knockout plants, but not in wild-type plants (-1.4-fold downregulated compared to efl18-treated Col-0). This gene encodes a cytosolic FPGS (FPGS3) that adds polyglutamate tails to folate and folate derivatives. A T-DNA line knocking out expression of this gene (Salk_038762C) is less responsive to a nonsaturating concentration of flg22, as measured by seedling growth inhibition. This *fpgs3* knockout line is also deficient in flg22-induced callose deposition. These plants also displayed fewer stained callose deposits than wild-type seedlings (Fig 7).

T-DNA lines knocking out the other *FPGS* genes encoded by the Arabidopsis genome were also studied. Two separate alleles of both *fpgs1* and *fpgs3* knockout plants produced statistically significantly more callose in response to flg22 than Col-0 or *fpgs2* knockout plants (Fig 7C). Additionally, the *fpgs1-2* (SALK_032544C) and *fpgs2-2* (SALK_821517C) alleles displayed statistically significantly less seedling growth inhibition than wild-type, *fpgs1-1*, and *fpgs2-1* plants (Fig 6E).

### Knocking out expression of an F-box gene, a TIR-domain containing gene, and a pectin methylesterase inhibitor disrupts flg22-induced callose deposition

At5g15660 (Salk_063563c, Salk_047400c) was identified from the microarray as a gene whose flg22-induced upregulation is broken by 3AB treatment (-3.5-fold downregulated compared to flg22-treated Col-0 plants). These knockout plants had fewer callose depositions than wild-type plants (Fig 7).

The gene At5g64640 encodes a pectin methylesterase inhibitor protein. This gene was identified through our microarray analysis as a *parg1-2* misregulated gene whose expression is only downregulated by elf18 in *parg1* knockout plants, but not in wild-type plants (-1.3-fold downregulated compared to elf18-treated Col-0 plants). A single At5g64640 knockout allele (Salk_063593c) shows attenuated responses to flg22 in the form of both reduced seedling growth inhibition (Fig 6) and fewer callose depositions (Fig 7). This seedling growth inhibition phenotype was not repeatable in a second allele (SALK_121275C), or at higher, saturating flg22 concentrations. Salk_063593c plants also had fewer callose depositions than wildtype (Fig 7).

The gene At1g47370 encodes a TIR (Toll-Interleukin-like Receptor) domain-containing protein. This gene was identified through our microarray as being upregulated by elf18 only in the *parg1* knockout background (2.2-fold upregulated compared to elf18-treated Col-0 plants). At1g47370 knockout plants (Salk_037091 and Salk_024124), show wild-type seedling growth inhibition (Fig 6), but are deficient in flg22-induced callose deposition (Fig 7). In preliminary studies all of the above-described knockout plants showed wild-type ROS burst and defense gene induction in response to flg22.

## Discussion

Gene expression profiling studies provide a number of opportunities for further characterization of molecular pathways of interest. For example, microarray data can be used to identify genes and acquire knockouts for a targeted mutant screen, to examine *cis*-regulatory element overrepresentation or Gene Ontology term overrepresentation, to conduct other pathway/biological process analyses, to compare with other published microarrays for common trends, and to manually examine expression patterns displayed by particular genes of interest [69–71].

The present transcriptomics study has opened up a number of research opportunities, some new and some previously implicated, for further study of the molecular mechanisms of both poly(ADP-ribosyl)ation and plant innate immunity and the links between them. We previously hypothesized putative roles for poly(ADP-ribosyl)ation in a subset of innate immune responses, including MAMP-regulated cell wall modifications, phenylalanine ammonia lyase activity, and genotoxic stress responses (Adams-Phillips 2008; Adams-Phillips 2010). This study was designed to investigate these hypotheses and provide a finer focus on how PARP, PARG and poly(ADP-ribose) may affect basal defense responses in plants. The following sections of this discussion address these hypotheses, as well as other new insights gained.

### PARP and PARG activity in the absence of MAMP exposure

A general examination of mRNA abundances across all treatments provided insights into the roles of PARP and PARG in untreated, unstressed plants. In this study, hierarchical clustering placed the overall transcriptome of 3AB-treated plants closest to untreated *parg1* mutants and wild-type plants (Fig 2), and in our hands 3AB-treated plants have appeared healthy (present study and Adams-Phillips 2008). However, we did identify genes that exhibit differential mRNA abundance between untreated and 3AB-treated Col-0 plants. Future study of those genes may offer insights into roles of PARP activity in relatively unstressed plants. The data from this study are available for other inquiries (S5 Table And GEO (http://www.ncbi.nlm.nih.gov/geo) #GSE100205). For example, although representing potentially problematic differences in mode/timescale of pathway inhibition, comparison of 3AB-treated plants with *parg1* plants might provide some insight into the transcriptional effect of depletion vs. accumulation of polyADP-ribose. The genome-wide hierarchical clustering presented in Fig 2 also demonstrates why further analysis of 3MB treatment data was not pursued - 3MB treatment caused a large number of mRNA abundance changes, suggesting pleiotropy and off-target effects of that PARP inhibitor. 3AB is likely to also have off-target impacts beyond its demonstrated inhibition of PARP, but apparently those impacts are much more limited.

3AB-treated plants did have reduced abundances of eight NB-LRR genes associated with plant defenses, and these PARP-inhibited plants also showed differential abundances of the transcripts of programmed cell-death and abiotic stress-related genes such as *GrimReaper (GRI*, At1g53130), *Syntaxin of Plants (SYP122* At3g52400), two DNAJ heat shock genes (At1g58725 and At3g13310), a heat stress transcription factor (*HSFA7A*, At3g51910), and the sulfur-deficiency related genes *LSU3* (At3g49570), *LSU1* (At3g49580), and *ATSDI1* (At5g48850).

Hierarchical clustering placed the overall transcriptome of *parg1* mutants closest to 3AB-treated plants (Fig 2), which itself is noteworthy. However, close inspection of their respective lists of genes differentially expressed relative to wild-type plants revealed an intriguing defense-related observation. Ten defense-related NB-LRR genes exhibited increased rather than decreased mRNA abundance in the *parg1* mutant. The de-activation of these genes in PARP inhibitor-treated plants and their reciprocal activation in *parg1* knockout plants suggests further possible impacts of plant poly(ADP-ribosyl)ation on plant immune responses. This type of observation also demonstrated the need for the subtraction scheme used above (Fig 3) to generate lists of genes whose mRNA abundances were truly affected by both MAMP treatment and either 3AB or *parg1* knockout.

### Use of GO term overrepresentation analysis as a discovery tool

Unlike many other transcriptomics studies [11] GO term overrepresentation analysis yielded few results that motivated us to further investigation in the current study. The fact that a “loosening” of statistical criteria was required in order to perform a such an analysis may partially explain this—most of the genes in the lists were only 1.2-fold up or downregulated, with q < 0.1. We therefore focused more on targeted knockouts and manual examination of gene lists. However, over-represented GO terms for this gene expression study included "chloroplast membrane", "chloroplast thylakoid membrane" and "photosystem" for flg22/3AB plants, suggesting possible stress points that arise when MAMP responses are disrupted by PARP inhibition. The "innate immune response" GO term was significant for elf18/*parg1*, bringing attention to specific elf18-activated defense-associated genes whose expression is altered in *parg1* plants. As with all microarray studies, only a limited set of conditions could be analyzed in the current study. We chose to focus our analysis on the plant response to flg22 and elf18 peptides at one hour after treatment, but it is likely that other time points and/or other pathogen-associated stimuli could uncover additional relevant pathways. Other users with different viewpoints may also be able to exploit not only the specific genes on the gene lists of this study, but also the GO analyses, to help shape future investigations.

### Poly(ADP-ribosyl)ation regulates a subset of innate immune responses

We previously reported that ROS synthesis and induction of some early defense genes remained intact in the presence of 3AB while callose and lignin deposition and PAL activity were inhibited [7]. A wide array of gene products display altered mRNA abundances upon MAMP treatment (up to a quarter of the genome, in fact), but only a small subset of those MAMP-regulated transcriptional responses is altered by PARP inhibitor or *parg1* knockout. This observation supports our previous findings (Adams-Phillips 2010) that only some—not all—MAMP-regulated basal defense responses are altered by disrupting poly(ADP-ribosyl)ation.

PARP inhibition disrupted a number of MAMP-regulated transcriptional responses that involve cell wall-related genes, further demonstrating a role for poly(ADP-ribosyl)ation in defense-related cell wall reinforcement. In the presence of flg22, 3AB downregulated a synbindin ER to golgi transport gene (At5g02280). PARP inhibition also disrupted the flg22-regulated accumulation of an alpha-glucosidase (oligosaccharide metabolism) transcript (At1g24320) and, perhaps most interestingly, a glyoxal oxidase-related gene product necessary for the production H_2_O_2_ for ligninolytic peroxidases (i.e., lignin biosynthesis) (At3g57620). 3AB treatment also disrupted the flg22-induced downregulation of a cellulose synthase gene (CESA10, At2g25540). And while no defense-related cell wall phenotypes have yet been reported for *PARG1* knockout plants, *PARG1* knockout did disrupt elf18-regulated upregulation of a pectate lyase transcript (At2g02720) while downregulating a pectin methylesterase inhibitor gene (At5g64640).

Poly(ADP-ribosyl)ation may also regulate the innate immune response via FPGS enzymes, specifically *FPGS3* (At3g55630). *FPGS3* attaches glutamate residues to folate and folate derivatives, especially THF-10 [72]. This isoform of FPGS is found in the cytosol, the location in which purine nucleotide synthesis primarily occurs [73]. Purine nucleotide synthesis is necessary for poly(ADP-ribosyl)ation, as the ADP-ribose units contain adenine. Further connections of FPGS3 to the innate immune response are discussed below.

Our previous report of 3AB blocking MAMP-induced PAL activity is also supported by the current microarray study. Expression of an AMP-synthetase family gene involved in phenylpropanoid metabolism (At1g20490) was downregulated when 3AB was added to flg22 treatment. This gene could provide a target for further examination into the mechanism(s) by which poly(ADP-ribosyl)ation regulates defense-related PAL activity and phenylpropanoid pigment production.

### Poly(ADP-ribosyl)ation at an intersection between plant defenses and DNA repair

A role for poly(ADP-ribosyl)ation in plant genotoxic stress responses has been demonstrated [10,29,42,52,53]. Early induction of host DNA double-strand breaks after infection by plant pathogens has been demonstrated [27]. The reactive oxygen species (ROS) burst activated by plant immune systems in response to pathogens has also been proposed to induce DNA damage that the plant would have to protect against or repair. Therefore it is not surprising that some DNA repair and reduction-oxidation homeostasis-related genes were either broken or misregulated by 3AB treatment or *PARG1* knockout. PARP inhibition broke the flg22-induced upregulation of oxidoreductase (At3g13610), glutathione-S-transferase transcripts (At1g02940 and At1g78340), and the flg22-regulated downregulation of a glutaredoxin family gene involved in protein disulfide oxidoreductase activity (At5g39865). In the presence of elf18, *parg1* plants had reduced expression of *RAD51B* (a double-stranded DNA repair gene) (At2g28560) and *SWI1* (involved in sister chromatid cohesion and chromosome organization) (At5g51330). The previously reported observation that PARP inhibition leads plants toward unproductive, toxic outcomes after exposure to MAMP [12] may also reflect a need for intact poly(ADP-ribosyl)ation machinery to protect the host from its induced defenses. Future studies that quantify reduction-oxidation and energy homeostasis inside plant cells undergoing defense responses (with and without disruption of poly(ADP-ribosyl)ation) could provide further insight into this hypothesis.

### Knocking out *PARG1* expression disrupts defense gene expression

The expression levels of a number of defense genes were also altered by *PARG1* knockout. The elf18-induced downregulation of a gamma interferon responsive lysosomal thiol reductase (which, in animals, recruits immunity-related MHC complexes to the plasma membrane) (At4g12890) was broken in *parg1* plants. *PARG1* knockout also broke elf18 induction of a number of other genes (Table 3) that are not common to any one signaling pathway, suggesting that PARG activity may be required not for any one specific pathogen-stimulated pathway, but rather at many junctures. This result is consistent with our previously-published results that *PARG2* gene expression is strongly upregulated by a wide variety of defense-associated stimuli, including MAMPs, biotrophic bacteria, necrotrophic fungi, and in constitutive defense mutants (Adams-Phillips 2010), despite the fact that *parg2-1* knockout plants remain visibly healthy. Such generalized responses may indicate roles for PARG and poly(ADP-ribosyl)ation in the plant host’s protection mechanisms of protection from its own induced defenses.

### Different MAMPs activate separate, yet overlapping, basal defenses

For many years, flg22 and elf18 peptides have often been used interchangeably as elicitors of basal plant defenses. Recognized by the separate PRRs FLS2 and EFR, respectively, they have been suggested to elicit very similar sets of downstream responses. And while many microarray studies over the years have examined either flg22 or elf18 transcriptional responses [74–77], the present study examined responses to both peptides within the *same* microarray experiment. Our analysis indicates that in our experiment system, 1690 genes were regulated by just flg22, while only 207 genes were differentially regulated by elf18 alone. From this result it is clear that the two receptors activate some of the same pathways, but with very different individual behaviors. Another clue that such differences in MAMP-elicited defenses exist could also be seen in our previously reported studies [7,12], which guided the experimental setup for this transcriptomics investigation (some effects of 3AB and/or *PARG1* knockout on basal defenses were only observed for flg22 or elf18 and not the other). This was a major reason why two different MAMPs were used in the current investigation. More recent investigations with EFR and FLS2 have demonstrated specific elements of signaling that are more prominent for one of the two receptors [77–79]. FLS2 is an evolutionarily older protein than EFR that is much more broadly distributed across plant families [80]. This may account for the larger set of gene products regulated only by FLS2, if FLS2 has had more time to gain new and different functions in addition to the core set of basal defenses that it activates in common with EFR.

### Dissecting roles of poly(ADP-ribosyl)ation in basal defense responses

Screens of T-DNA knockouts chosen based on the gene lists generated in this study identified five candidate genes that may play a role in poly(ADP-ribosyl)ation’s impacts on basal defense responses. These knockouts were identified based on altered seedling growth inhibition and/or callose deposition phenotypes in response to flg22. We screened 135 Arabidopsis T-DNA lines and identified one showing altered seedling growth inhibition and four showing altered callose deposition phenotypes. The reduced growth inhibition and callose deposition suggest that the plants may not be properly detecting the presence of MAMP, or that their ability to respond to MAMP is compromised.

At3g55630 was identified from the microarray as a *parg1-2* misregulated gene whose expression is only downregulated by elf18 in *parg1* knockout plants, but not in wild-type plants. This observation suggests that PARG1 protein may be necessary for de-repression of *FPGS* expression in response to MAMP elicitation. This gene encodes a cytosolic folylpolyglutamate synthetase (*AtFPGS3*) that adds polyglutamate tails to folate co-enzymes, enhancing their stability and affinity [72]. A T-DNA line knocking out expression of this gene (Salk_038762c) is less responsive to 0.05 μM flg22, as measured by seedling growth inhibition, and callose deposition. Additionally, a T-DNA line knocking out expression of the FPGS1 isoform of FPGS (Salk_032544C) is also less responsive to flg22 than wild-type. These altered responses suggest that at least one folate-dependent enzyme—many of which are involved in methionine biosynthesis and photorespiration [72]—may be necessary for wild-type levels of basal defense response activation.

An intriguing link exists between folylpolyglutamylation and another pathway implicated by our knockout screens: pectin methylesterification. *fpgs3* single mutant plants have reduced levels of methionine and methylesterified pectins [72]. At5g64640 encodes a pectin methylesterase inhibitor that was downregulated by elf18 only in *parg1* knockout plants, displaying a similar expression pattern to *FPGS3*, and further implicating poly(ADP-ribosyl)ation in the regulation of MAMP-induced cell wall reinforcement. At5g64640 knockout plants show a reduced response to flg22 peptide, with fewer callose deposits compared to wild-type plants when treated with flg22. Pectinesterases are a group of cell-wall localized proteins that catalyze the de-esterification of methyl-esterified D-galactosiduronic acid units in cell wall pectins, creating acidic pectins that can lower cell wall pH, allowing cell expansion and growth, and leading to either cell wall stiffening (*via* production of pectate gels in the apoplasm) or cell wall loosening (*via* proton-stimulated activity of cell wall hydrolases) [81–83]. Pectinesterases are known to be involved such physiological responses as pollen tube growth and abscission. A pectin methylesterase inhibitor protein was demonstrated to be necessary for basal disease resistance and antifungal activity in pepper [84,85], but this enzyme’s role in plant defenses remains unclear. In this current study, the absence of *PARG1* led to downregulation of a pectin methylesterase inhibitor gene. Further studies will be required to characterize both the role that PARG1 plays in this pectin methylesterase inhibitor’s gene expression, as well as the function of this cell wall-associated enzyme in MAMP-induced basal defenses.

Beyond containing conserved F-box, there is little known about the other two genes identified in our basal defense screen, At5g15660 and At1g47370. F-box protein domains regulate protein-protein interactions, creating links between target proteins and ubiquitin-conjugating enzymes [86,87]. TIR-X proteins resemble TIR-NB-LRR resistance proteins, but lack the nucleotide binding and leucine-rich-repeat domains. When activated by pathogen effectors, resistance (R) proteins activate a wide range of defense responses, and TIR-X proteins have been hypothesized to modulate the activity of some TIR-NB-LRR proteins [88,89]. RBA1 (At1g47370), the TIR-X protein identified in our screen, has recently been shown to induce cell death in response to the pathogen effector HopBA1.

In summary, disruption of poly(ADP-ribosyl)ation processes alters the expression of a number of plant genes during the early stages of the response to the MAMPs flagellin (flg22) and EF-Tu (elf18). Knowledge of the specific genes that are impacted can contribute to the generation of hypotheses for future research regarding poly(ADP-ribosyl)ation and/or more general aspects of plant responses to pathogens.

## Supporting information

S1 Table Normalized calls for gene lists (relaxed). (XLSX)

S2 Table Normalized calls for gene lists (stringent). (XLSX)

S3 Table Normalized calls for flg22 vs. elf18 gene lists. (XLSX)

S4 Table T-DNA screen overview. (XLSX)

S5 Table Full array data: Abundance values for each treatment replicate for all 60,770 gene probes, as well as average normalized call values and fold-change versus untreated Col-0. (XLSX)

S6 Table Expression levels of defense genes regulated by flg22 versus untreated. (XLSX)
